# *Citrobacter* spp. bloodstream infection primarily affects the elderly either hospitalized or closely associated with health care – a population-based observational study with comparisons between *C. koseri* and the *C. freundii* complex

**DOI:** 10.1186/s12879-025-11972-6

**Published:** 2025-10-20

**Authors:** Torgny Sunnerhagen, Hiba Taie, Johan Ohlsson, Oskar Ljungquist

**Affiliations:** 1 Division of Infection Medicine, Department of Clinical Sciences, Lund University, Lund, Sweden; 2 Department of Clinical Microbiology, Skåne University Hospital, Region Skåne, Lund, Sweden; 3 Department of Infectious Diseases, Helsingborg Hospital, Helsingborg, Sweden

**Keywords:** Bacteraemia, Antimicrobial resistance, Incidence, Mortality, Bacterial infection, Blood culture

## Abstract

**Objectives:**

Despite regularly being found in blood cultures, there are few studies of bloodstream infection (BSI) with *Citrobacter.* In this population-based study, the aim was to explore patient characteristics, outcome, and incidence in a publicly funded single payer setting.

**Methods:**

Patients with growth of *Citrobacter* in blood culture were identified through the clinical microbiology laboratory in Lund, Sweden, for the year 2013–2023. This laboratory serves the whole Skåne region, with a population of 1.4 million. Medical records were retrospectively reviewed.

**Results:**

554 episodes of *Citrobacter* BSI were identified, with septic shock seen in 25 (4%) episodes and with 38 (7%) episodes resulting in intensive care. The 90-day mortality was 18% and the median age of the patients was 77 years. Resistance to cephalosporins was below 10%, and carbapenem resistance was not found in any isolates. A majority of episodes were healthcare associated or nosocomial, and almost half of BSI cases originated from the urinary tract. The association with the urinary tract was stronger for *C. koseri* BSI than for *C. freundii* complex BSI, which was more often polymicrobial and associated with abdominal surgery.

**Conclusion:**

*Citrobacter* BSI was primarily found in elderly patients, either hospitalized or in close contact with healthcare in other ways. *C. koseri* BSI was to a greater extent associated with urinary tract focus compared to patients with BSI due to *C. freundii* complex, which was associated with abdominal source of infection and polymicrobial BSI.

**Supplementary Information:**

The online version contains supplementary material available at 10.1186/s12879-025-11972-6.

## Introduction

*Citrobacter* are genus of Gram-negative bacteria in the order Enterobacterales, named for their ability to use citrate as a carbon source. Facultatively anaerobic, *Citrobacter* species are found both in the environment and in the human gastrointestinal tract [1–3]. *Citrobacter* spp. are recognized for their involvement in a diverse range of infections affecting the blood and various other sites, including the urinary tract, liver, biliary tract, peritoneum, intestines, bone, respiratory tract, endocardium, wounds, soft tissues, and meninges [4–6]. There are at least nineteen known species within the *Citrobacter* family, and it can be challenging to correctly classify species in routine clinical microbiology [7, 8].

In a recent study of blood culture positivity, *Citrobacter* species represented 0.66% of positive blood cultures, placing it as less common than the fungus *Candida* but more common than *Haemophilus* or *Serratia* [9]. Previous studies focusing on bloodstream infection (BSI) caused by *Citrobacter* are few, but a common theme is that the patients generally suffered from comorbidities (most commonly disease of the abdomen) [10–12]. This is something that is also seen in studies investigating *Citrobacter* infection with other foci, where hospital transmission is also frequently reported.

Carbapenemase production due to acquisition of mobile genetic elements is also an increasing concern in some settings [13–17].

Population-based incidence and trend estimates describing *Citrobacter* bloodstream infections (CBSI) during recent years are few. To be able to work with preventive measures, it is important to monitor CBSI trends and rates of hospital acquired bloodstream infections. In this population-based study, the goal was to comprehensively explore the incidence, trend, clinical outcome and patient characteristics of *Citrobacter* BSI in a population-based setting with a publicly funded single-payer health care service, consisting of both university hospitals and smaller hospitals.

## Methods

### Study design, setting, population

This was an observational, population-based, retrospective study between years 2013–2021 in Skåne, southern Sweden. Patients with *Citrobacter* in blood culture were identified by searching the laboratory information system of Clinical Microbiology, Region Skåne. This university hospital laboratory serves all clinics and hospitals in the region, both private and public. Patients whose medical records were unavailable were excluded.

### Microbiological methods

During the study period, BACTEC FX (BectonDickinson) was the blood culture system used, with the Bactec Lytic anaerobic medium and Bactec Aerobic Plus medium. A standard blood culture consisted of one aerobic and one anaerobic bottle. Susceptibility testing was performed by disk diffusion according to the European Committee on Antimicrobial Susceptibility Testing (EUCAST) standards [18]. For species identification, matrix-assisted laser desorption/ionization time-of-flight mass spectrometry (MALDI-TOF MS: Bruker Daltonics, using the Bruker MBT Compass library version most recent at the time of sample analysis) was used as the main method for species identification. To separate the *Citrobacter freundii* complex (*C. freundii/braaki/gillenii/murliniae/rodentium/sedlakii/werkmannii/youngae*) from *C. koseri*, colour of colonies on CHROMagar (CHROMAgar) and indole tests were used in addition to the MALDI-TOF MS results.

### Data sources

Data was retrieved from Clinical Microbiology of Region Skåne, in Lund. This department processes all clinical microbiology samples in the region. Medical records were reviewed using the electronic health records system Melior (Melior, Siemens Healthcare Services, Upplands Väsby, Sweden), by using a predefined study protocol with variables of interest.

### Definitions and variables

Charlson Comorbidity Index (CCI) was used to assess comorbidity. Immunosuppression was defined as a patient with previous organ transplantation, or ongoing immunosuppressive medication, such as TNF-alpha inhibitor or corticosteroid treatment exceeding 15 mg prednisolone per day, or previous stem cell transplantation, or primary immune defect, or ongoing or recently terminated cancer-treatment such as chemotherapy, dialysis or severe chronic kidney disease or ongoing treatment for autoimmune disease. Polymicrobial blood culture was defined as the presence of a pathogen other than *Citrobacter* in a blood culture. For patients with more than one episode of BSI, the first episode was used to report age, CCI etc. for individual patients. Only one *Citrobacter* episode per hospitalization was considered in the incidence calculations.

Primary bloodstream infection was defined as no other source of *Citrobacter* spp. was found during medical records review [19]. Disease severity was assessed using National Early Warning Score (NEWS) [20]. Community-acquired, nosocomial and health care associated infection were defined according to a previously published definition [21]. The Sepsis 3 criteria were used to define septic shock [22].

### Statistical analysis

Crude incidences rates (IR) were determined by dividing the number of CBSI episodes by the population of Skåne. To enable comparison and to adjust for demographic changes over the study time, IR were age- and sex-standardized to the 2021 population of Skåne, reported as episodes per 100,000 person-years with 95% confidence interval (CI). A regression model using the joinpoint trend analysis software (https://surveillance.cancer.gov/joinpoint/) was constructed to analyse trends of IR during the study period, expressed as annual percentage change (APC) with 95% CI. The Kaplan-Meier estimator, with the log rank test to detect statistically significant differences, was used to estimate survival between patients with bloodstream infections due to *C. freundii* complex and *C. koseri*. Only the first episode was included in the analysis. For unevenly distributed continuous data, the Mann–Whitney U test was used and for categorical data the Chi^2^ test was used. Univariate and multivariate models were constructed based on the first *Citrobacter* BSI episode. In the adjusted model, putative variables plausible to be associated with 90-day mortality were chosen and reported with odds ratios with 95% confidence intervals. A p-value less than 0.05 was considered statistically significant.

## Results

### BSI episodes and baseline characteristics

During the study period, there were 554 episodes of *Citrobacter* spp. BSI in 525 patients. In total, eighteen patients each experienced two episodes of CBSI, four patients had three episodes of CBSI and one patient had four episodes of CBSI. The most common species was *Citrobacter freundii* complex (47%, *n* = 261), followed by *Citrobacter koseri* (42%, *n* = 233) and *Citrobacter non-freundi* complex, *non-koseri* (11%, *n* = 60). The median age of the included patients was 77 years (range 0-100), and 70% of the patients were men (*n* = 374). The median CCI was 6 (range 0–15) and 28% (*n* = 146) of patients were considered immunocompromised (Table 1). Out of all 534 episodes of CBSI, complete medical records were available for 537 episodes (97%).

**Table 1:** Baseline characteristics and clinical determinants of included patients. All percentages calculated on available data

Variables	*n* (%)
Age, y, median	77 (0-100)
Male sex	374 (71)
CCI, median (range)	6 (0–15)
Immunosuppression	146 (28)
Substance abuse	22 (4)
Duration of symptoms, median (range)	1 (0–28)
Urinary symptoms	172 (33)
Abdominal pain	166 (31)
Fever	387 (73)
Abdominal focus	95 (19)
Urinary focus	245 (48)
Total NEWS score, median, (range)	4 (0–18
Peripheral leucocyte count, 10^9^/L, median	12.9 (0.1–92)
CRP, mg/L, median	108 (< 4-563)
Lactate, mmol/L, median	2.4 (0.6–15)

### The incidence of *Citrobacter koseri* increased during the study period

**Fig. 1:**
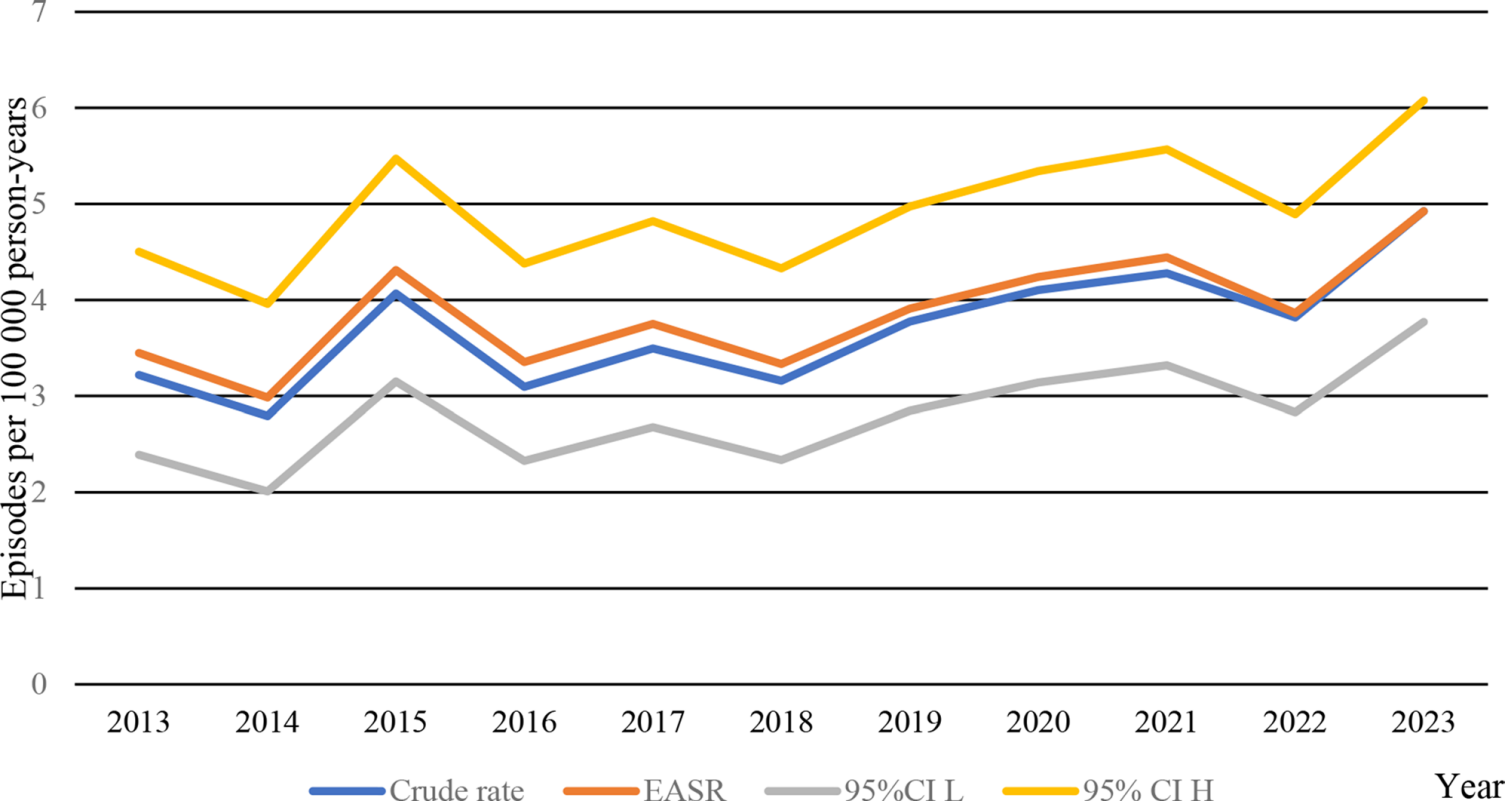
Incidence of *Citrobacter* spp. during the study period. EASR = estimated age- and sex standardized rate. CI L = Confidence Interval Low. CI H = Confidence Interval High

The age and sex-standardized incidence rate of *Citrobacter* spp. BSI increased from 3.4 episodes per 100 000 person-years in 2013 to 4.9 episodes per 100 000 person-years in 2023 (Fig. 1).

The increase was not statistically different from zero, with an APC of 3.2 (95% CI: -0.2% to 6.6%) for the entire study period. Separated into species, the crude rate of *Citrobacter koseri* and *Citrobacter freundi* complex increased from 0.9 to 1.9 in 2013, to 2.1 and 2.1 in 2023, respectively (Table A1). While the increase in crude incidence of *Citrobacter freundi* complex BSI was not statistically different from zero (APC of 2.74 95%CI: -2.3% to 8.1%), the crude incidence of increase of *Citrobacter koseri* during the study period was statistically significant (APC of 7.56 95%CI: 4.6% to 10.6%). Most BSI occurred in ages 75–84 years (Figure A1).

### Microbiological findings and antimicrobial resistance

Resistance rates were statistically significantly higher for *Citrobacter freundii* compared to *Citrobacter koseri* for all antimicrobials compared, apart from gentamicin (Table A2). For *Citrobacter freundii*, resistance rates were 15% (*n* = 40) for ceftazidime, 7% (*n* = 18) for ciprofloxacin, 11% (*n* = 28) for piperacillin-tazobactam, 8% (*n* = 20) for trimethoprim/sulfamethoxazole and 1% (*n* = 6) and 2% (*n* = 9) were resistant against gentamicin and tobramycin, respectively. For *Citrobacter koseri*, 0.4% (*n* = 1) strains were resistant towards ciprofloxacin, 2% (*n* = 4) towards piperacillin-tazobactam and 1% (*n* = 2) towards trimethoprim/sulfamethoxazole. No strains were resistant towards ceftazidime, gentamicin or tobramycin. No carbapenem-resistant strains were found in the study.

In total, 38% (*n* = 208) of the BSI episodes were polymicrobial, with 306 pathogens other than *Citrobacter* detected. The most common microbiological findings in polymicrobial blood cultures other than *Citrobacter* spp. were *Enterococcus* spp. (32%, *n* = 66), *Klebsiella* spp. (25%, *n* = 51) and *Escherichia coli* (24%, *n* = 49) (Table 2).

**Table 2:** Microbiological findings in polymicrobial BSI other than *Citrobacter* species

Pathogen	*C. freundii* complex *n* (%)	*C. koseri* *n* (%)	*Citrobacter non-freundi* complex, *non-koseri n* (%)
*Enterococcus* spp.	38 (21)	16 (24)	12 (2)
*Klebsiella* spp.	41 (23)	1 (1)	9 (15)
*Escherichia coli*	20 (11)	21 (31)	8 (13)
*Streptococcus* spp.	15 (8)	6 (9)	1 (2)
*Staphylococcus* spp.	11 (6)	6 (9)	5 (8)
*Proteus* spp.	5 (3)	8 (12)	11 (18)
*Clostridium* spp.	6 (3)	4 (6)	3 (5)
*Enterobacter* spp.	10 (6)	0 (0)	4 (7)
*Pseudomonas aeruginosa*	10 (6)	1 (1)	0 (0)
Other	23 (13)	4 (6)	7 (12)

### Citrobacter bloodstream infection is most often associated with urinary tract infection

**Fig. 2:**
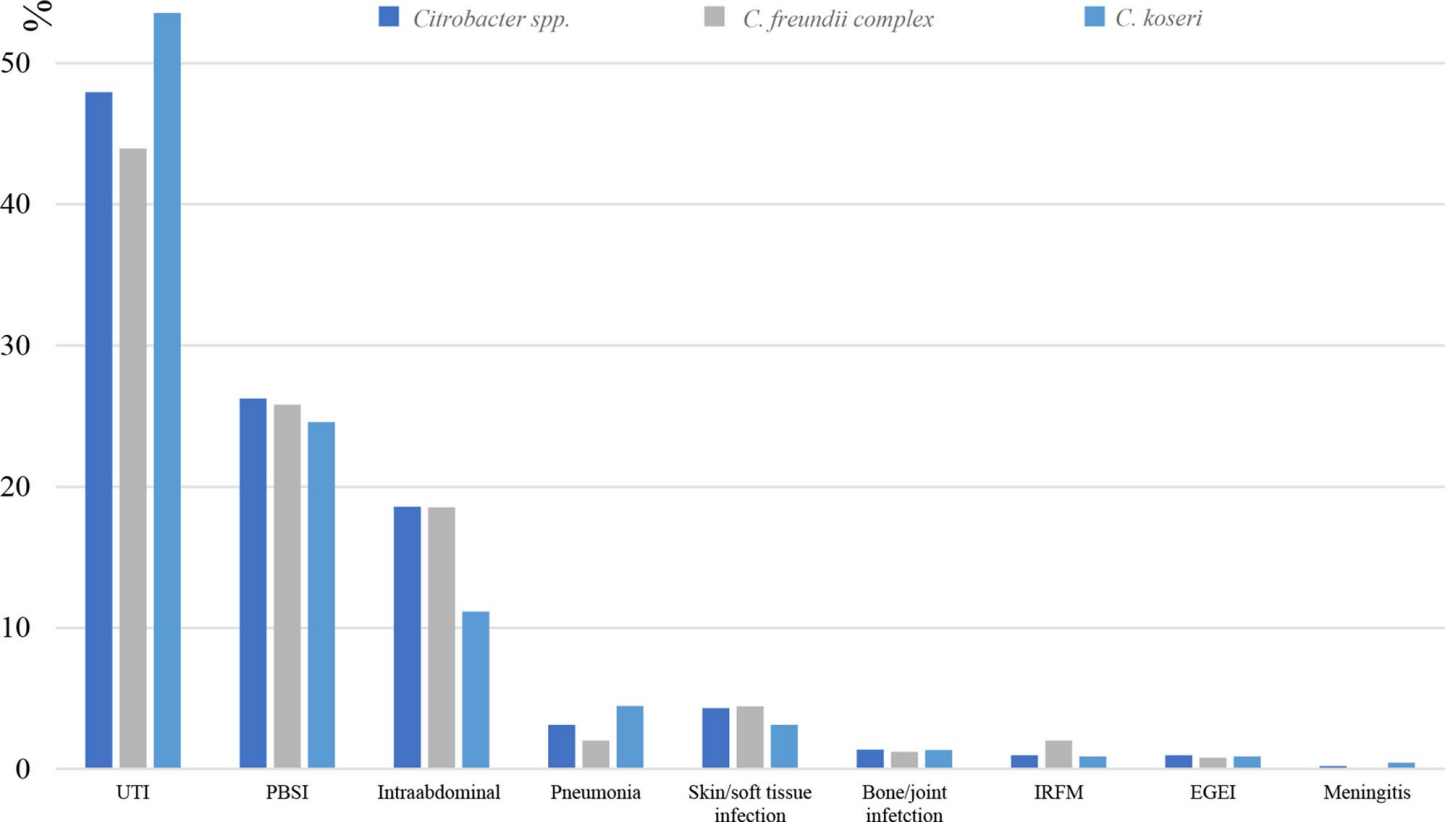
Infection sources for *Citrobacter* BSI. UTI = urinary tract infection. PBSI = primary bloodstream infection. IRFM = infection related to a foreign material (portacath, central venous catheter or orthopaedic prosthesis). EGEI = endocarditis/graft/endograft infection

According to the medical records, the most common etiology of *Citrobacter* BSI was urinary tract infection 48% (*n* = 245). Urine cultures were obtained in 404 (73%) of BSI episodes, of which 373 (92%) were acquired prior to antimicrobials had been initiated. Out of all 404 urine cultures, 187 (46%) were positive for *Citrobacter* spp. Primary BSI constituted 24% (*n* = 134) of all *Citrobacter* BSI, after which intraabdominal source of infection was common 19% (*n* = 95) (Fig. 2).

### Citrobacter bloodstream infection is associated with healthcare

**Fig. 3:**
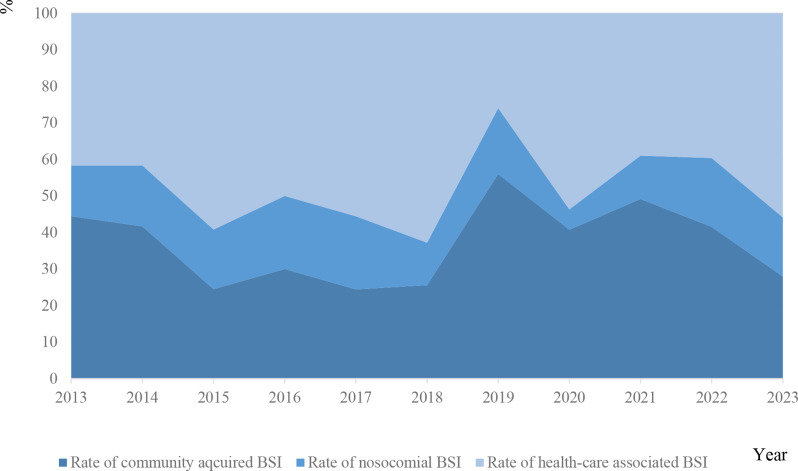
Mode of acquisition of *Citrobacter* BSI

In total, fever at blood culturing or within 48 h was present for 387 (70%) episodes and patients reported shivers in 252 (46%) episodes. The median NEWS score when blood cultures were obtained was 4 (range 0–18), the median CRP value was 108 mg/L (range < 4-563) and the median leukocyte count in blood was 12.9 10^9^/L (0.1–92) (Table 1). In nine episodes (2%) of *Citrobacter* BSI, patients were managed as outpatients, whereas hospitalization was required for all other BSI episodes. In total, 197 (36%) *Citrobacter* BSI were community acquired, 81 (15%) episodes were nosocomial and 255 (46%) were health care associated (Fig. 3). The median length of stay at hospital was 8 days (range 0-680).

### Outcome

**Fig. 4:**
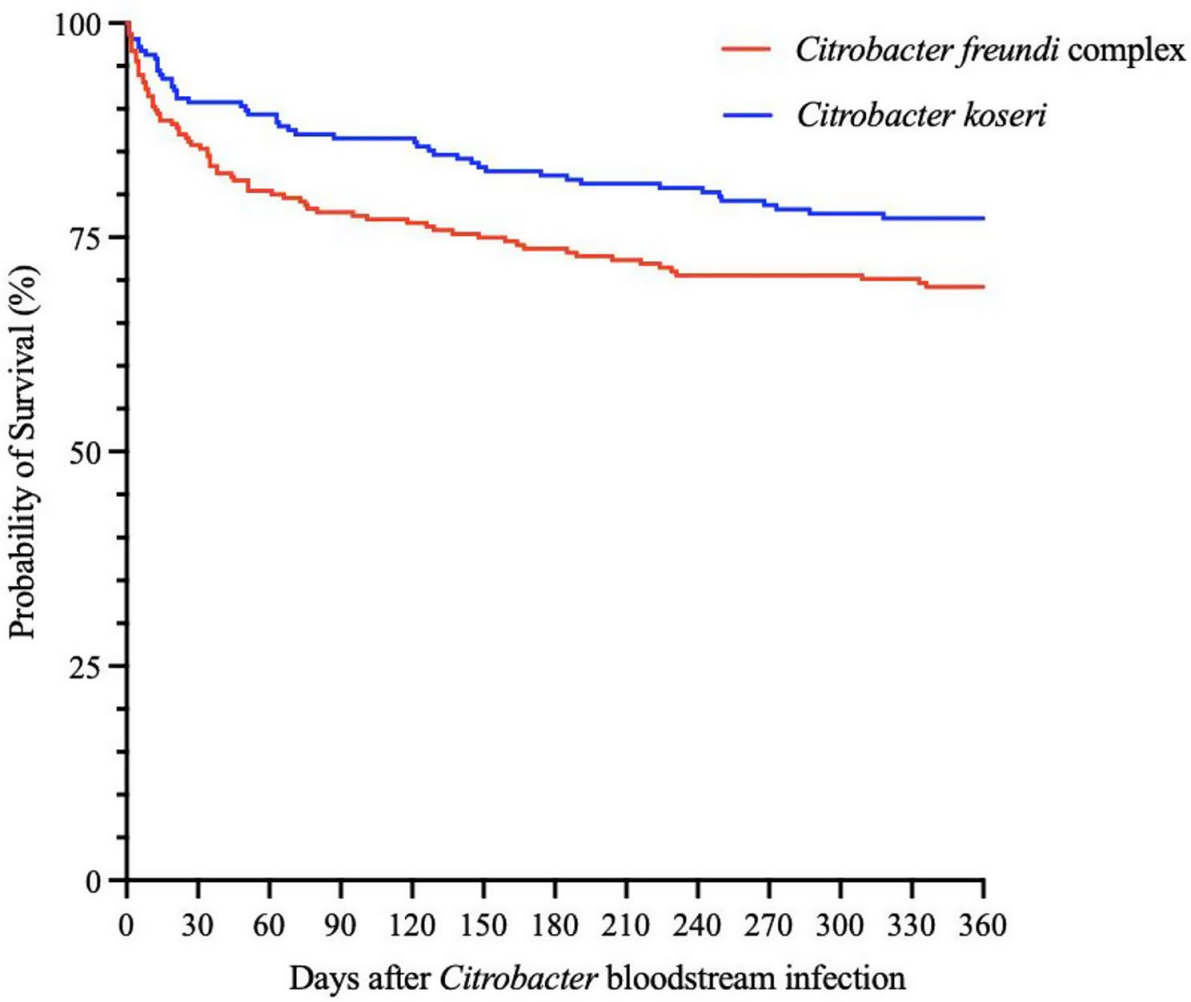
Kaplan-Maier estimates of mortality in *C. freundii* complex and *C. koseri* BSI. *p* = 0.09

Out of all 554 *Citrobacter* BSI episodes, septic shock was seen in 25 (4%) episodes and in 38 (7%) episodes patients required intensive care. The 30-, 90-, 180- and 365-days mortality rates were 12%, 18%, 23% and 25%, respectively (Table 3). Although the 90-, 180- and 365-day mortality rates were significantly higher for *C. freundii-*complex BSI compared to *C. koseri* BSI, there was no statistically significant difference in mortality rates between *C. freundii* complex BSI compared to *C. koseri* BSI in the Kaplan-Meier estimator (Fig. 4). There was no statistical difference in all-cause mortality within 90 days between patients with one episode of *Citrobacter* spp. BSI (19%) and patients with two or more episodes (22%, *p* = 0.74).

**Table 3:** Outcome of *Citrobacter* spp. BSI. ICU = intensive care unit

Variables	*n* (%)
Septic shock	25 (5)
Length of stay, days, median (range)	8 (0-680)
Intensive care treatment	38 (7)
Length of stay at ICU, days, median (range)	2 (0–71)
Death within 30 days	65 (12%)
Death within 90 days	101 (18%)
Death within 180 days	127 (23%)
Death within 365 days	141 (25%)

### Differences in patient characteristics between BSI due to *C. freundii* complex and *C. koseri*

There were no differences in age, CCI-score, rate of immunosuppression or substance abuse between patients with BSI caused by *C. freundii* complex and *C. koseri.* However, a greater proportion of men suffered from *C. koseri* BSI compared to *C. freundii-*complex BSI (*p* = 0.01). The rate of polymicrobial BSI (*p* = 0.0001) as well as BSIs with abdominal source of infection (*p* = 0.03) were greater for *C. freundii* complex compared to BSIs due to *C. koseri*. Patients with *C. koseri* BSI had to a greater extent UTI symptoms (*p* = 0.005**)**, positive nitrite dipsticks (*p* = 0.006**)**, and analogous *Citrobacter* species cultured in the urine (*p* = < 0.0001) compared to patients with BSI due to *C. freundii* complex (Table 4).

**Table 4:** Differences in clinical characteristics between patients with *C. freundii* complex and *C. koseri*

	*C. freundii* complex, *n* = 251 (%)	*C. koseri*,* n* = 216 (%)	*p*-value
Age, y, median	77	77	0.71
Male sex	169 (68)	168 (78)	**0.01**
CCI score, median	6	6	0.098
Immunosuppression	69	54	0.3751
Substance abuse	10 (4)	10 (5)	0.76
Polymicrobial BSI	116 (46)	54 (25)	**< 0.0001**
*Citrobacter* in urine culture	67 (33)	102 (53)	**< 0.0001**
Hospitalized	234	208	0.99
Length of stay, median	9	7	0.0196
Need of intensive care	17 (7)	15 (7)	0.97
Length of stay at ICU, median	4	2	0.1995
Death within 30 days	36 (14)	20 (9)	0.09
Death within 90 days	56 (22)	29 (13)	**0.01**
Death within 180 days	65 (26)	38 (18)	**0.03**
Death within 365 days	71 (28)	43 (20)	**0.0356**
Community acquired CBSI	80 (34)	88 (42)	0.07
UTI symptoms	68	88	**0.005**
Positive nitrite dipstick	29 (24)	43 (41)	**0.006**
Abdominal pain	79 (33)	53 (25)	0.06
Temperature > 38 within 48 h of culture	166 (70)	160 (76)	0.1
Shivers	108 (46)	107 (51)	0.27
Abdominal focus	46 (19)	25 (11)	**0.03**
Urinary focus	109 (46)	120 (57)	0.019
Primary BSI	64 (27)	55 (26)	0.84
Pneumonia	5 (2)	10 (5)	0.12
Systolic blood pressure, mmHg, median	120	126	0.0299
Pulse, beats per minutes, median	95	95	0.82
Respiratory rate	20	20	0.99
RLS level, median	1	1	0.8
Saturation, median	96	96	0.43
Temperature, median	38.1	38.1	0.43
Total NEWS, median	4	4	0.47
Radiology performed of abdomen/urinary tract	118 (50)	88 (92)	0.095
Septic shock	12 (5)	9 (4)	0.715
Peripheral leucocyte count, 10^9^/L, median	13.1	13	0.48
CRP, mg/L, median	104	113	0.24
Lactate, mmol/L, median	2.7	2.3	0.087

RLS = reaction level scale. ICU = intensive care unit. CCI = Charlson Comorbidity Index. CRP = c-reactive protein. NEWS = national early warning score. UTI = urinary tract infection. CBSI = *Citrobacter* bloodstream infection. BSI = bloodstream infection. Includes patient characteristics of the first episode with each species

### Univariate and multivariate analysis of variables associated with mortality within 90 days

In the univariate model, female sex, higher CCI score, immunosuppression and longer duration of hospitalisation was associated with death within 90 days of *Citrobacter* BSI, as was lack of UTI symptoms and non-community acquired infection (Table 5).Similarly, primary BSI and abdominal focus was significantly more common in patients that died within 90 days. In the multivariate model, higher Charlson comorbidity index, immunosuppression, higher CRP, and a urinary tract focus, were independently associated with 90 days mortality, while age, sex, septic shock, polymicrobial culture and fever were not (Table 6).

**Table 5:** Univariate logistic regression model

	Mortality within 90 days *n* = 96 (%)	Survived > 90 days *n* = 429 (%)	*p*-value
Age, y, median	76	77	0.694
Male sex	58 (60)	315 (74)	**0.0089**
CCI score, median	8	5	**< 0**,**0001**
Immunosuppression	46 (48)	99 (24)	**< 0**,**0001**
Substance abuse	5 (5)	18 (4)	0,6613
Polymicrobial BSI	45 (47)	156 (36)	0.0555
*Citrobacter* in urine culture	20 (38)	157 (42)	0.4912
Hospitalized	95 (99)	410 (98)	0.5423
Length of stay, median	14	7	**< 0.0001**
Need of intensive care	4 (4)	32 (7)	0.2485
Length of stay at ICU, median	5	2	0.24
Community acquired CBSI	23 (24)	165 (40)	**0.0027**
UTI symptoms	21 (22)	147 (36)	0.**0088**
Positive nitrite dipstick	7 (18)	58 (27)	0.2658
Abdominal pain	35 (37)	121 (30)	0.1823
Temperature > 38 within 48 h of culture	60 (64)	308 (76)	**0.0171**
Shivers	38 (40)	201 (50)	0.1076
Abdominal focus	23 (24)	68 (17)	0.1014
Urinary focus	21 (22)	209 (52)	**< 0.0001**
Primary BSI	40 (30)	93 (23)	**0.0002**
Pneumonia	16 (17)	9 (2)	**< 0.0001**
Systolic blood pressure. mmHg, median	115	126	**0.0164**
Pulse. beats per minutes, median	95	96	0.6492
Respiratory rate, median	22	20	0.0934
RLS level, median	1	1	0.0766
Saturation, median	96	96	0.8484
Temperature, median	37.8	38.2	**0.0418**
Total NEWS, median	5	4	**0.0415**
Radiology performed of abdomen/urinary tract	51 (53)	182 (45)	0.1426
Septic shock	5 (5)	19 (5)	0.8173
Peripheral leucocyte count. 10^9^/L, median	12.4	13	0.6794
CRP. mg/L, median	102	132.5	**0.0195**
Lactate. mmol/L, median	2.7	2.3	**0.0119**

RLS = reaction level scale. ICU = intensive care unit. CCI = Charlson Comorbidity Index. CRP = c-reactive protein. NEWS = national early warning score. UTI = urinary tract infection. CBSI = *Citrobacter* bloodstream infection. BSI = bloodstream infection. Includes patient characteristics of the first episode with each species

**Table 6:** Multivariate analysis of variables associated with mortality within 90 days

Variable	Odds ratio	95% Confidence Interval	*p*-value
Age	1.008	0.9868 to 1.031	0.47612
CCI	1.229	1.114 to 1.361	**< 0.0001**
Sex (ref: male)	1.479	0.8614 to 2.519	0.1515
Immunocompromised	2.225	1.282 to 3.860	**0.0043**
Septic shock	1.683	0.5094 to 4.758	0.3525
CRP	1.003	1.000 to 1.005	**0.0441**
Polymicrobial BSI	1.0115	0.6620 to 1.864	0.6798
Fever	0.6348	0.3741 to 1.087	0.0940
UTI	0.3513	0.1930 to 0.6222	**0.0004**

CCI = Charlson Comorbidity Index. CRP = c-reactive protein. NEWS = national early warning score. UTI = urinary tract infection

## Discussion

We aimed to comprehensively study *Citrobacter* spp. bloodstream infection in our population-based setting in southern Sweden. During the eleven years studied, we found that *Citrobacter* spp. BSI primary affected the elderly, who were either hospitalized or closely associated with health care. The age- and sex-standardized incidence of *Citrobacter* spp. BSI increased during the study period, although not statistically different from zero. However, while the crude incidence of *C. freundii* complex BSI remained stable, we observed a crude incidence of *C. koseri* BSI that increased significantly. Given that the proportion of elderly is expected to rise in the coming decades, this finding warrants careful attention and ongoing surveillance.

Though a direct comparison to other studies is difficult, owing to differences in testing methodology and antimicrobials included, the rate of antimicrobial resistance against ceftazidime and piperacillin/tazobactam was low [5, 10, 11] and no carbapenem-resistant isolates were discovered. We found higher rates of antimicrobial resistance in general for *C. freundii* compared to *C. koseri*, in line with previous findings [8, 23].

The literature is scarce with population-based estimates of *Citrobacter* spp. bloodstream infections. A recent review article reported a non-population-based pooled incidence of *Citrobacter spp.* BSI of 0.175 episodes per 1000 patients during the study period, which are difficult to compare to our results [14]. The increasing trend of Citrobacter BSI in our study is consistent with the results of our previous study in our region, that revealed a general increase of BSI [9]. Most severe community-acquired bacterial infections saw a marked decrease during the covid-19 pandemic, a trend that was not seen in our setting for *Citrobacter* spp. BSI [24]. Instead, hospital-acquired BSI increased during the covid-19 pandemic, and the increasing incidence of Citrobacter spp. BSI could be explained by the high and increasing rate of hospital acquired and healthcare related during 2020-23 (Fig. 4) [25]. A high rate of nosocomial *Citrobacter* BSI has previously been reported [7, 26].

Our study reveals that *Citrobacter spp.* BSI primarily affects the elderly, with most episodes occurring in ages 75–85 years. Furthermore, our results suggests that *C. koseri* BSI more often originates from the urinary tract as compared to *C. freundii* complex BSI, which is more often associated with polymicrobial growth and intra-abdominal source of infection. In our study, the mortality rate was higher in the long run (within 90, 180 and 365 days), but not within 30 days, for *C. freundii* compared to *C. koseri.* A previous study found that carriage of the bla_TEM−1_ resistance gene was an independent risk factor for 28-day mortality in *Citrobacter* BSI. While our study demonstrated substantially lower antimicrobial resistance rates compared to this previous research, a causative association between the increased resistance rates observed in *C. freundii* compared to *C. koseri* and the higher mortality rates seen for *C. freundii* remains plausible and warrants investigation in future prospective studies. Additionally, our findings that *C. freundii* infections were more frequently associated with polymicrobial growth and intra-abdominal sources may also contribute to the observed higher mortality rates in this species complex [4].

As the proportion of elderly is expected to rise, the incidence of *Citrobacter spp.* will likely continue to increase in the years to come. Healthcare workers need to be prepared to face *Citrobacter spp.* bloodstream infections with antimicrobial resistant strains could be problematic, particularly for elderly individuals with comorbidities.

Our study has several strengths, including its population-based design with complete capture of *Citrobacter* spp. BSI cases across the Skåne healthcare region, comprehensive longitudinal follow-up and a substantial cohort of patients. Limitations include potential selection biases inherent to retrospective cohort studies, such as regarding the identification of comorbidities, and missing data. Another limitation is the lack of molecular characterization, which prevents us from reporting crucial epidemiological data regarding clonal relationships, transmission patterns, and specific resistance mechanisms among the isolated *Citrobacter* strains, such as differentiating plasmid transmitted and chromosomal AmpC-production.

To conclude, in this population-based observational study, *Citrobacter* spp. bloodstream infection primary affected elderly, either hospitalized or closely associated with health care. The crude incidence of *C. koseri* increased significantly over the study period, whereas the crude incidence of C. *freundii* complex remained stable. *C. koseri* BSI was to a greater extent associated with urinary tract focus compared to patients with BSI due to *C. freundii* complex, which was associated with abdominal source of infection and polymicrobial BSI.

## Supplementary Information

Below is the link to the electronic supplementary material.

Supplementary Material 1

## Data Availability

Anonymized data is available upon reasonable request to the authors.
