# Supplementary material for: Citrobacter spp. bloodstream infection primarily affects the elderly either hospitalized or closely associated with health care – a population-based observational study with comparisons between C. koseri and the C. freundii complex
Source: BMC Infect Dis. 2025 Oct 20;25:1353. doi: 10.1186/s12879-025-11972-6 (PMC12538971; doi:10.1186/s12879-025-11972-6)
Supplement: Supplementary file 1 — Supplementary Material 1 [file 12879_2025_11972_MOESM1_ESM.docx]

| **Year** | **Crude rate** | **SASR** | **95%CI L** | **95% CI H** | ***Citrobacter koseri*** | ***Citrobacter freundii* complex** |
| --- | --- | --- | --- | --- | --- | --- |
| 2013 | 3.22 | 3.45 | 2.39 | 4.50 | 0.94 | 1.88 |
| 2014 | 2.79 | 2.98 | 2.01 | 3.96 | 0.93 | 1.16 |
| 2015 | 4.07 | 4.31 | 3.15 | 5.47 | 1.46 | 1.84 |
| 2016 | 3.10 | 3.35 | 2.33 | 4.38 | 1.51 | 1.51 |
| 2017 | 3.50 | 3.75 | 2.68 | 4.82 | 1.56 | 1.71 |
| 2018 | 3.16 | 3.33 | 2.34 | 4.33 | 1.39 | 1.69 |
| 2019 | 3.77 | 3.91 | 2.85 | 4.97 | 1.74 | 1.52 |
| 2020 | 4.10 | 4.24 | 3.14 | 5.34 | 1.51 | 2.02 |
| 2021 | 4.28 | 4.44 | 3.32 | 5.57 | 1.93 | 1.93 |
| 2022 | 3.82 | 3.86 | 2.83 | 4.89 | 1.91 | 1.63 |
| 2023 | 4.92 | 4.92 | 3.77 | 6.08 | 2.18 | 2.32 |
| Mean | 3.70 | 3.87 | 2.80 | 4.94 | 1.55 | 1.75 |

**Table A1**. Yearly incidence of *Citrobacter* spp*., Citrobacter koseri* and *Citrobacter freundii* complex incidence rates per 100,000 person-years. SASR = sex and age specific incidence rate. CI L = confidence interval low**.** CI H = confidence interval high

**Figure A1.** BSI episodes related to age intervals.

| **Antibiotics** | ***Citrobacter freundii* complex*,* resistant strains *n (%)*** | ***Citrobacter koseri***  **resistant strains *n (%)*** | ***p* -value** |
| --- | --- | --- | --- |
| Ceftazidime | 40 (15) | 0 (0) | <0.0001 |
| Ciprofloxacin | 18 (7) | 1 (0.4) | 0.0005 |
| Piperacillin-tazobactam | 20 (8) | 2 (1) | 0.0001 |
| Trimethoprim/sulfamethoxazole | 6 (1) | 2 (1) | 0.0006 |
| Tobramycin | 9 (2) | 0 (0) | 0.0116 |
| Gentamicin | 6 (1) | 0 (0) | 0.0552 |

**Table A2. Comparisons of resistance rates of *Citrobacter freundii and Citrobacter koseri***
